# Female sterility: a new strategy for hybrid rice production

**DOI:** 10.1093/plphys/kiag160

**Published:** 2026-03-30

**Authors:** Jessy Silva

**Affiliations:** Assistant Features Editor, Plant Physiology, American Society of Plant Biologists; LAQV/REQUIMTE, Department of Biology, Faculty of Sciences, University of Porto, Porto, Portugal

Plant reproduction is essential for human sustenance, with grains and fruits comprising over 90% of global agricultural production. Seeds provide the majority of calories consumed by humans, including staple cereals such as wheat, rice, and maize (Khoury et al. 2022). In flowering plants, seeds arise from sexual reproduction. The pistil, the female reproductive organ, receives pollen on its stigma, where pollen germinates to form a pollen tube that transports the male gametes to the ovule. Within the ovule, male and female gametes fuse in a process called double fertilization, producing a new seed containing the embryo and nutritive endosperm (Yadegari and Drews 2004).

Rice (*Oryza sativa*) is a staple food for more than half of the world's population (Visakh et al. 2024); therefore, increasing its yield is a major goal in agriculture. Typically, rice self-fertilizes, with pollen from the anther landing directly on the stigma of the same flower. However, when 2 different rice varieties are crossed, the resulting progeny (hybrid seeds) often exhibit hybrid vigor, producing higher yields than either parental plant. In conventional hybrid rice production, breeders use pollen from a normal plant (paternal plant) to fertilize a male-sterile plant (maternal plant), which cannot produce pollen but can produce seeds. After pollination, both parental plants produce seeds, so breeders must manually remove the paternal plants to ensure only hybrid seeds from the maternal plant are harvested—a process that is slow, labor-intensive, and expensive. One solution is to use female-sterile plants as the pollen donor (paternal plant). Female-sterile plants produce pollen but cannot form seeds, allowing them to fertilize male-sterile plants while preventing unwanted seed production and facilitating mechanical harvesting of hybrid seeds (Zeng et al. 2017; Li et al. 2022). Therefore, developing female-sterile rice lines is essential to reduce labor and accelerate hybrid rice breeding.

In this issue of *Plant Physiology*, Li et al. (2026) report the isolation of a female-sterile rice mutant from a γ-irradiation–induced mutant library. Although the mutant exhibited normal vegetative growth and produced viable pollen, it displayed multiple stigmas and defective female gametophytes (embryo sacs) caused by persistent nucellus—ovule tissue that surrounds the embryo sac and normally degenerates as the embryo sac matures—preventing seed formation. The authors named this mutant *defective pistil with multiple stigmas 1* (*Osdpms1*) and employed map-based cloning to identify the gene responsible for the phenotype, uncovering a large chromosomal deletion consistent with γ-irradiation mutagenesis. Using the identified DNA sequence, they generated a CRISPR/Cas9-edited mutant, which presented the same female-sterile phenotype. Complementation of the mutant with the *OsDPMS1* gene partially restored fertility, confirming that a single gene within the deleted region corresponds to the *OsDPMS1* gene.

Through a bioinformatic analysis, Li and colleagues found that OsDPMS1 protein contained WD40 repeat domains, which are known to mediate protein interaction (Schapira et al. 2017).

Using protein-protein interaction assays, such as yeast 2-hybrid, in vitro GST pulldown, and bimolecular fluorescence complementation in *Nicotiana benthamiana* heterologous system, the authors revealed that OsDPMS1 interacts with 3 proteins, OsUBQ, OsCDC48, and OsCDC48E, which are involved in the ubiquitin-proteasome degradation pathway. For example, CDC48 is an ATPase Associated with Diverse Cellular Activity (AAA-type ATPase) that binds polyubiquitinated proteins and facilitates their delivery to the proteasome for degradation (Bègue et al. 2019). Mutations in *OsCDC48* cause premature senescence and lethality, highlighting its essential role in protein homeostasis (Huang et al. 2016).

Based on these findings, the authors propose a model in which OsDPMS1 regulates pistil morphogenesis and female gametophyte development by controlling protein stability through the ubiquitin–proteasome pathway. In wild-type plants, OsDPMS1 forms a complex with OsCDC48 and OsCDC48E that marks regulatory proteins—responsible for pistil primordia proliferation and nucellar cell survival—for degradation via the ubiquitin–proteasome pathway. This controlled protein degradation ensures proper stigma number and triggers nucellar programmed cell death, providing space and nutrients for the developing embryo sac. In *Osdpms1* mutant, this complex fails to form, leaving the regulatory proteins active. As a result, extra stigmas develop, nucellar cells persist, and embryo sac formation is disrupted, leading to female sterility.

Female-sterile plants are valuable for hybrid rice production, but because they cannot produce seeds, special strategies are required to propagate them. To overcome this limitation, Li and colleagues generated a transgenic maintainer line for *Osdpms1* by adapting the seed production technology (SPT) system originally developed in maize to maintain male-sterile lines (Wu et al. 2016). In the *Osdpms1* mutant, the authors introduced a transgenic SPT construct containing the *OsDPMS1* gene to restore fertility, a pollen α-amylase that depletes starch and prevents pollen germination, and a red fluorescent seed marker to identify transgenic seeds (Fig. 1a). The resulting maintainer line is hemizygous (carrying only 1 copy) for the SPT construct; thus, half of the pollen grains do not carry the SPT transgene and can fertilize ovules, while the other half carry the transgene and cannot fertilize plants, preventing inheritance of the SPT transgene via pollen (Fig. 1a). Consequently, SPT transgenes are passed through only the female parent. Self-pollination of the maintainer line by nontransgenic pollen produces 2 types of seeds: 50% of nontransgenic yellow seeds that are female-sterile seeds and ready for hybrid breeding, and 50% of red fluorescent seeds carrying the SPT transgene, which can be detected by fluorescence and removed (Fig. 1a). Therefore, the SPT system allows propagation of sterile plants without spreading the transgene, ensuring that the female-sterile seeds used for hybrid crosses remain nontransgenic (Wu et al. 2016).

**Figure 1:**
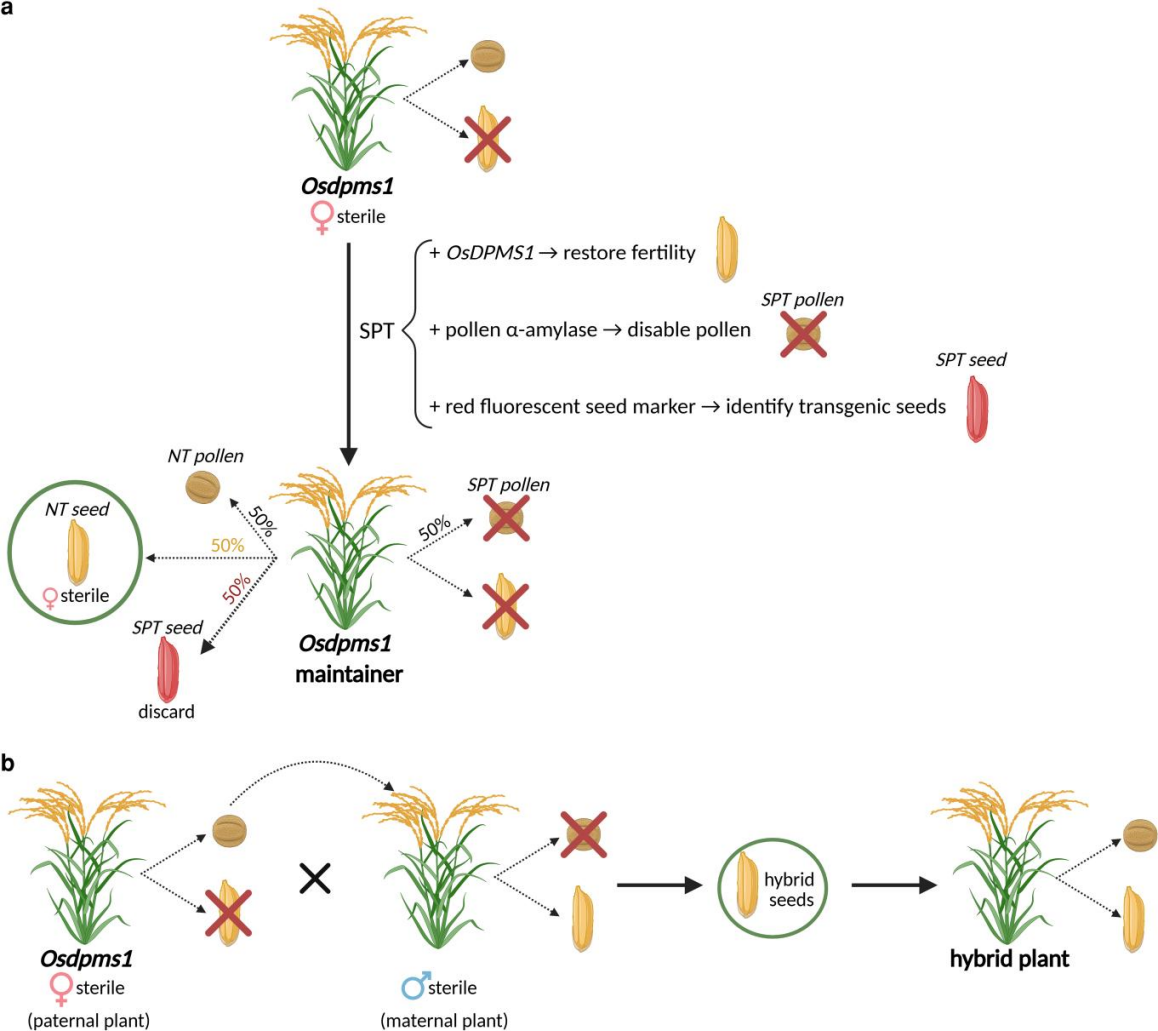
Hybrid rice seed production using female-sterile *Osdpms1* plants. **a)** Propagation of female-sterile *Osdpms1* using a maintainer line via the SPT approach. The *Osdpms1* maintainer line carries the SPT transgene, which includes the *OsDPMS1* gene to restore fertility, a pollen α-amylase to disable transgenic pollen (SPT pollen) by depleting starch, and a red fluorescent seed marker identifying transgenic seeds (SPT seeds). During self-pollination, SPT pollen (50%, black font) is nonfunctional, while nontransgenic pollen (NT pollen; 50%, black font) can fertilize ovules, producing 2 types of seeds: nontransgenic and female-sterile seeds (NT seeds; 50%, yellow font) for hybrid crosses, and SPT seeds (50%, red font) identified by red fluorescence and removed. **b)** Hybrid seed production using *Osdpms1*. Female-sterile plants (*Osdpms1*) serve as pollen donors (paternal plants) to fertilize male-sterile plants (maternal plants). Fertilization generates hybrid seeds that carry genetic contributions from both parents, which grow into hybrid plants exhibiting hybrid vigor. Created in https://BioRender.com.

Beyond revealing a molecular mechanism controlling pistil morphogenesis and female gametophyte development via OsDPMS1 and the ubiquitin–proteasome pathway, Li et al. also provide a practical strategy for hybrid rice breeding. By developing *Osdpms1* maintainer lines, the authors enabled efficient propagation of female-sterile plants, which can serve as pollen donors to fertilize male-sterile maternal lines, allowing efficient hybrid seed production without manual removal of paternal plants (Fig. 1b). Future studies should identify the regulatory proteins targeted by OsDPMS1, clarify how proteasome-mediated degradation shapes pistil patterning, and determine whether similar mechanisms operate in other crops, potentially expanding the tools available for hybrid seed production.

## Recent research articles in *Plant Physiology*:

- Gu and Han (2024) summarized recent progress in understanding the genetic basis of hybrid vigor and highlighted emerging approaches for intelligent hybrid rice breeding (https://doi.org/10.1093/plphys/kiae385).
- Lu et al. (2024) reported that delayed expression of the MYB transcription factor MOF1 represses tapetal genes, impairing anther function and producing male-sterile lines that can facilitate polyploid hybrid rice breeding (https://doi.org/10.1093/plphys/kiae145).

## Data Availability

No new data were generated or analyzed in support of this research.
